# Unusual trivial trauma may end with extrusion of a well-functioning penile prosthesis: a case report

**DOI:** 10.1186/1752-1947-1-34

**Published:** 2007-06-27

**Authors:** Nader Salama, Tomoteru Kishimoto, Hiro-Omi Kanayama, Susumu Kagawa

**Affiliations:** 1 Departments of Urology, Alexandria School of Medicine, Alexandria, Egypt; 2 Tokushima School of Medicine, Tokushima City, Japan

## Abstract

**Background:**

Diabetes mellitus (DM) is the most common indication for insertion of a penile prosthesis and is a risk factor for infection of such prostheses.

**Case presentation:**

Two patients presented with infected prostheses following unusual trivial penile trauma. Both patients underwent exploration and removal of the prostheses with uneventful recovery.

**Conclusion:**

Appropriate sizing of the prosthesis should be taken into account to ensure good concealment and avoid easy exposure of the penis to unexpected trauma. Use of the newly designed antibiotic-coated prostheses appears preferable. As soon as signs of prosthesis infection appeared, extrusion of the device should be expedited.

## Background

Penile prostheses continue to be required even in the era of newly available oral medications. These prostheses can be either semirigid or hydraulic. Implantation of a semirigid prosthesis is relatively straightforward with a low complication rate and offers effective treatment of erectile dysfunction that has been unresponsive to pharmaco- therapy. Significant benefits to quality of life have been reported for both patients and their partners [1]. DM is the most common indication for prosthesis implantation and also represents a risk factor for prosthesis infection [1].

This report describes the cases of two patients who experienced unusual trivial penile trauma resulting in infection and ultimately extrusion of a successfully inserted and well functioning penile prosthesis.

## Case presentation

### Case 1

A 57-year-old man was admitted to our clinic with a history of fever and pain, erythema and swelling of the penis. He had undergone placement of a Mentor Acuform penile prosthesis (13 mm) 18 months earlier. He claimed the prosthesis had been functioning well, giving him and his two wives, as he had a polygamous marriage, an excellent degrees of satisfaction [1]. He had a 20-years history of DM (type II) but the disease was under control. He also reported having bumped his penis into the suitcase of the preceding passenger while boarding an airplane five days prior to presentation.

### Case 2

A 64-year-old man was admitted to our clinic with similar complaints. He had undergone placement of the same type of penile prosthesis three years earlier. He reported the prosthesis had been functioning well, providing a high degree of satisfaction for him and his wife [1]. He had a 17-year history of DM (type II) with good control. He also described having trapped his penis against a toilet seat while sitting down to defecate four days earlier.

**Figure 1:**
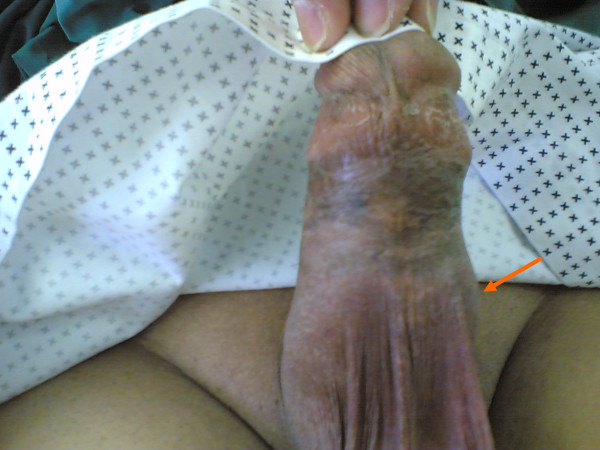
Appearance of the penis on initial examination in Case 1. The arrow shows edema on the peno-scrotal junction.

At presentation, both patients displayed fever (38.6°C and 39°C, respectively), and reported receiving broad spectrum antibiotics from general practitioners in their home towns. They denied any previous similar episodes since prostheses implantation. Physical examination in both cases revealed an erythematous, edematous and indurated penis with mildly macerated skin. The first patient also had ischemic spots over the penile shaft and localized soft swelling (3 × 2.5 cm) on the left side of the peno-scrotal junction (Fig. 1). Penile and perineal palpation indicated intact devices in place, and this was further confirmed by radiography of the pelvis. However, the appearance of the patients' organs looked abnormal with poor concealment of the devices. The patients' white blood counts were elevated (13.200/mL and 14,100/mL, respectively). Urine analyses and cultures yielded negative results. Diabetes was well controlled in both patients as evidenced by normal levels of fasting and postprandial blood sugars and glycosylated hemoglobin levels. Ultrasound examination of the genitalia was performed to identify any possible hematoma but yielded no relevant results other than edema at the peno-scrotal junction of the first patient. Blood examination for bleeding, coagulation, prothrombin and partial thromboplastin times yielded normal results.

After discussion in each case, we decided to perform an exploration and extrusion of each penile prosthesis. The operations were performed under spinal anesthesia. The tunica was opened and a significant volume of whitish-yellow purulent material was noted around both cylinders of the device in both patients. Cultures of this material in both patients yielded positive results for *Staphylococcus epidermidis *(*S. epidermidis*). The localized swelling seen at the peno-scrotal junction of the first patient was confirmed to represent soft tissue edema but not hematoma. Removal of the prostheses followed by continuous irrigation and suction drainage resulted in rapid and complete resolution of the local inflammatory process and infection-associated symptoms within three to four days and recovery was uneventful in both cases.

## Discussion

Penile prosthesis infection has been reported in many studies with an incidence of about 8.9 %, mostly occurring in the first year postoperatively [2]. DM is prominent in the etiology of erectile dysfunction and has also been a feature of most cases of penile prosthesis infection [1,2]. Problems in neurovascular, immune and micro-circulatory systems are well-known to be associated with DM [3], and may contribute to the higher rate of prosthesis infection in diabetic patients.

In the present report, *S. epidermidis *was isolated on culture taken from the explored wounds of both patients. This supports the findings of several studies showing *S. epidermidis *as the most common organism found at removal of penile prostheses due to infection [4]. *S. epidermidis *is present in all portions of the body living within the superficial layers of the epidermis surrounded by the biofilm; a protective coating [5]. Given the symbiotic nature of this bacteria living outside the immunological system of the body it incites little immunological response when it is the cause of infection related to a prosthesis. Patients infected with this organism may remain asymptomatic for long periods. These bacteria probably arrived in the prosthesis as surgical contaminants during the initial surgery [6].

Both patients, in the current report, had penile prostheses with 13 mm diameter. These prostheses are somewhat bulky and cannot be satisfactorily crammed into relatively small organs nor allow for complete concealment. This lack of appropriate concealment might facilitate easier exposure of patient organs to unexpected trauma, as evidenced by the soft tissue edema in the first patient. Although the trauma reported in these cases appeared trivial it may have been sufficient to break up the biofilm generated by the offending organism; at least partially, with detachment and dispersal of the organism in a planktonic fashion leading to rapid progression of the infection process. This is consistent with the findings of Costerton et al [7] who showed that trauma could represent a potential triggering events for disengagement of bacterial biofilms. In support of our explanation, two lines of evidence were present. First, the isolated organism was *S. epidermidis*. The biofilm made by this bacterium is formed of multiple cell layers resting on the biomaterial surface and protected by an amorphous slimy material [8]. This slime is not a true capsule, but is loosely bound to the staphylococcal cells. This slime may be less resistant to shearing force during washing or during trauma causing its break up [9]. Second, both patients were diabetic and several in-vitro studies have showed that glucose and its analogues, although inducing the formation *of S. epidermidis *biofilm, also distinctly inhibit its strong attachment to biomaterials [10].

However, this trivial trauma induced only minimal biofilm detachment and the antibiotics, therefore, received by our patients were thus ineffective to stop the impending infection. This suggestion agrees with several previous studies proposing that if the biofilm is not sufficiently damaged, antibiotic diffusion into the periprosthetic area will be hindered making the antibiotic concentration significantly lower compared to the level in serum [11]. Several episodes of such trivial trauma might have affected each of our patients since they underwent implantation surgery. Nevertheless, they passed un-noticed as they were not associated with the signs of significant inflammation related to the reported trauma that made these occasions memorable.

On the other hand, the chronic pressure exerted by the cylinders of these 13 mm prostheses with subsequent tissue ischemia, and in presence of DM with its well known microcirculatory and immuno-compromising problems [3], could provide a good environment for prosthesis infection to occur. While most reported penile prosthesis infections occur in the early post-operative period [2], late infections have been also documented. A review of the literature revealed late prosthesis infection due to hematogenous seeding from significant remote entry sites, in the absence of trauma, including patients with active Crohn's disease, skin ulcers and dental abscesses. However, these reports involved only a small number of patients and so they appeared to occur infrequently although the infection was obvious when it occurred [12]. Our two cases appear to represent the first reported instances of late-onset prosthesis infection precipitated by trivial accidental trauma in the absence of any demonstrable source of infection.

## Conclusion

When the implantation of a malleable penile prosthesis is considered, appropriate sizing should be taken into account to ensure good concealment and to allow the patient to avoid easy exposure of the penis to unexpected trauma. Patients with such prostheses should also be carefully instructed about the importance of concealing the device. New antibiotic-coated prostheses should be considered for insertion particularly in patients with conditions such as diabetes to decrease the subsequent risk of device infection. Device extrusion should be expedited as soon as signs of prosthesis infection appear, since antibiotic use alone is likely to be of little value.

## Competing interests

The author(s) declare that they have no competing interests.

## Authors' contributions

The contributing authors made a critical review of this manuscript.
